# Handrub dispensers per acute care hospital bed: a study to develop a new minimum standard

**DOI:** 10.1186/s13756-021-00949-0

**Published:** 2021-06-16

**Authors:** Sabine Kuster, Jan A. Roth, Reno Frei, Christoph A. Meier, Marc Dangel, Andreas F. Widmer

**Affiliations:** 1 Division of Infectious Diseases and Hospital Epidemiology, University Hospital Basel, University of Basel, Petersgraben 4, 4031 Basel, Switzerland; 2 Medical Director, University Hospital Basel, University of Basel, Petersgraben 4, 4031 Basel, Switzerland; 3 Present Address: Department of Internal Medicine, Clinic and Amublance of Internal Medicine, University Hospital Zurich, University of Zurich, Rämistrasse 100, 8091 Zurich, Switzerland; 4 Swissnoso, Swiss National Center for Infenction Prevention, Sulgenecstrasse 35, 3007 Bern, Switzerland

**Keywords:** Hand hygiene, Alcohol-based handrub dispensers, Location, Quality standards

## Abstract

**Background:**

Accessibility to alcohol-based handrub (ABHR) dispenser is crucial to improve compliance to hand hygiene (HH), being offered as wall-mounted dispensers (ABHR-Ds), and/or pocket bottles. Nevertheless, information on the distribution and density of ABHR-Ds and their impact on HH have hardly been studied. Institutions such as the World Health Organisation or the Centers for Disease Control and Prevention do not provide guidance. The Robert-Koch-Institute (RKI) from Germany recommends an overall density of > 0.5 dispensers per patient bed. We aimed to investigate current conditions in hospitals to develop a standard on the minimal number of ABHR-D.

**Methods:**

Between 07 and 09/2019, we applied a questionnaire to 178 hospitals participating in the Swissnoso National Surveillance Network to evaluate number and location of ABHR-Ds per bed in acute care hospitals, and compared the data with consumption and compliance with HH.

**Results:**

110 of the 178 (62%) hospitals provided data representing approximately 20,000 hospital beds. 83% hospitals provided information on both the total number of ABHR-Ds and patient beds, with a mean of 2.4 ABHR-Ds per bed (range, 0.4–22.1). While most hospitals (84%) had dispensers located at the room entrance, 47% reported also locations near or at the bed. Additionally, pocket-sized dispensers (100 mL) are available in 97% of hospitals.

**Conclusions:**

Swiss hospitals provide 2.4 dispensers per bed, much more than governmental recommendation. The first study on the number of ABHR-Ds in hospitals may help to define a minimal standard for national and international recommendations

**Supplementary Information:**

The online version contains supplementary material available at 10.1186/s13756-021-00949-0.

## Background

Hand hygiene (HH) using alcohol-based handrub (ABHR) belongs to the standard measures to prevent transmission of pathogens. Alcohol-based handrub dispensers (ABHR-Ds) at the point-of-care are recommended by the World Health Organization (WHO) [1]. However, data on the number and best location for ABHR-Ds in hospitals wards are scarce. The German Office of Public Health (Robert Koch Institute, RKI) recommends at least one dispenser per bed in intensive care units and 0.5 dispenser per bed in general wards [2]. In contrast, the WHO, the Centres for Disease Control and Prevention (CDC) and the European Centre for Disease Prevention and Control (ECDC) do not provide specific recommendations on the number of ABHR-Ds to optimize HH compliance.

Several studies highlighted that HH compliance may improve if the ABHR-Ds are visible and easily accessible, whereas standardized locations have no significant impact [3–5]. Hence, the ABHR consumption depends on the hospital type (higher in university hospitals) but also on the availability of ABHR-Ds on the wards [6, 7] Consistent with this finding, adding dispensers on a medical ward lead to an increase in HH events; two dispensers per bed were considered to be optimal [8]. Various factors can influence HH compliance including ABHR-D location, teaching and promotion of hygiene measures and time-saving applications (ABHR vs. hand washing with soap) [9–14]. Regardless of whether ABHR is provided by pocket-sized dispensers or permanently mounted dispensers, accessibility plays the key role in HH compliance [15) However, the optimal location may also depend on the workflow in a patient’s room and the preferences of the healthcare workers [16]. Additionally, the location within the point-of-care area has more impact on ABHR consumption than the number of ABHR-Ds in room [17, 18]. A mathematical model was even proposed to estimate optimal locations of ABHR-Ds in hospital wards [19].

Hospitals and patient rooms are not standardized; therefore, it is difficult to provide general recommendations on the optimal number and locations for ABHR-Ds. However, a minimum of ABHR-Ds could be defined to set a standard in hospitals to improve compliance by easy access. This study aimed to investigate the current number and locations of ABHR-Ds in Swiss acute care hospitals as basis for setting national minimal standards on the number of required ABHR-Ds.

## Methods

Between July and September 2019, we applied an anonymous, standardized questionnaire on HH practices as well as on the number of ABHR-Ds per bed and location to all 178 hospitals participating in the Swissnoso National Surveillance Network. Swissnoso was founded in 1994, to establish guidelines to prevent nosocomial infections and evolved as National Centre for Infection Prevention (www.swissnoso.ch). Around 80% of all the 268 acute care hospitals participate in the network, including in surveillance of surgical site infections, urinary tract infections, as well as compliance of HH, and antibiotic stewardship.

Our questionnaire (see Additional file 1) was addressed to board-certified infection control practitioners and hospital epidemiologists, using an electronic as well as paper-based tool (TeleForm, Electric Paper (Schweiz) GmbH, Lachen, Schweiz. We adapted our questionnaire following the outline used in the PROHIBIT (Prevention of Hospital Infections by Intervention and Training) study, whose questionnaire was validated for a Europe-wide survey in 2010 [7]. All hospitals participating in Swissnoso received an invitation to participate in our online survey via a central mailing list. Duplicates were identified by an identical number of patient beds and hospitalisation numbers, as well as the unique—but anonymous -identification number. The availability of pocket dispensers was based on self-declaration; the participant hospitals were asked to estimate the percentage of pocket dispenser use in relation to total ABHR consumption After the questionnaire was evaluated for technical feasibility at the University Hospital Basel, participant hospitals were asked to provide information on the number of ABHR-Ds per patient room, the total number of ABHR-Ds in the hospital, availability of pocket dispensers, the locations of ABHR-Ds within the patient room and the consumption of ABHR in litres per year. Non-responders were reminded at least twice before being considered as definite non-responders. The primary outcome was the mean number of ABHR-Ds per room and per hospital bed as well as the most common locations of ABHR-Ds in Swiss hospitals. In a secondary analysis, we investigated the correlation between the total number of ABHR-Ds per hospital bed and ABHR consumption (in mL) per patient day; the analysis was stratified by hospital size. Of note, in Switzerland even small hospitals may have a certified intensive-care unit (ICU) with at least six hospital beds, therefore we did not specifically asses the number and locations of ABHR-Ds on different ICUs compared to general wards.

Statistical analyses were performed with Stata/IC, version 15 (Stata Corp LLC, Texas, United States of America). To explore correlations between hand-rub consumption and the mean number of dispensers on a hospital level, Spearman’s rank correlation coefficients were calculated. A two-sided *p*-value of < 0.05 was considered significant.

## Results

During the three-month study period, 110 of 178 (62%) participating hospitals provided data, representing approximately 20,000 acute care hospital beds in Switzerland. Sixty percent (n = 66) of hospitals reported more than 100 hospital beds. Table 1 summarizes data on location and number of ABHR-Ds, pocket dispenser use and HH compliance monitoring in different hospital categories. Most frequently ABHR-Ds are placed at the entrance of the room (n = 92, 84%), followed by dispensers near the sink (n = 81, 74%). In total, 47% (n = 52) reported an ABHR-D location near or at the bed. Other frequently mentioned locations of ABHR-Ds were mobile devices such as computer trolleys.

**Table 1:** Location and number of dispensers depending on hospital size

	All hospitals (n = 110)	Hospitals with < 200 beds (n = 70)	Hospitals with 200–500 beds (n = 30)	Hospitals with > 500 beds (n = 10)
Location of ABHR* dispensers				
Entrance (%)	92 (84)	58 (83)	26 (87)	7 (70)
Near the sink (%)	81 (74)	47 (67)	25 (83)	9 (90)
Within 1 m-radius from the bed (%)	31 (28)	19 (27)	5 (17)	7 (70)
At bedside (%)	26 (24)	19 (27)	5 (17)	2 (20)
Elsewhere (for example trolleys) (%)	14 (13)	10 (14)	2 (7)	2 (20)
Number of mounted dispensers				
Per room, mean (SD, range)	1.8 (0.8; 1–4)	2 (0.8; 1–4)	1 (0.8; 1–4)	2 (1; 1–3)
Per bed, mean (SD, range)	2.4 (3; 0.4–22.2)	2.5 (3.6; 0.8–22.2)	2.1 (1; 0.7–5.3)	2.1 (1.5; 0.4–4.9)
Pocket dispenser use > 60%	28 (26)	14 (20)	10 (33)	4 (40)
HH* compliance monitoring	68 (62)	37 (53)	25 (83)	5 (50)

^*^ABHR Alcohol-based handrub, *HH* hand hygiene

Wall-mounted dispensers are the dominant type of ABHR dispensers in 75% (n = 82) of hospitals compared to 25% where pocket-sized dispensers are mainly offered. However, 97% (n = 107) hospitals provide pocket-sized dispensers as an additional tool to wall-mounted dispensers to facilitate access to handrub. Overall, the reported mean number was two ABHR-Ds per patient room (mean: 1.83, range 1–4). Out of 110 hospitals, 91 (83%) provided detailed data on the total number of beds and dispensers within the building, not only in patient rooms, resulting in a higher mean of 2.4 (range 0.4–22.1) ABHR-Ds per hospital bed. Therefore, the number of ABHR-Ds per bed is higher than the number of ABHR-Ds per room

When hospitals reported a mean number of two ABHR-Ds per room, the most common mentioned locations were at the entrance and near the sink (n = 69, 63%) followed by ABHR-Ds at the entrance and within one-meter radius of the bed (n = 20, 22%). Three ABHR-Ds per room—either at the entrance, near the sink and within one-meter radius of the bed, or at the entrance, near the sink, and at the foot end of the bed—were reported in 15% (n = 16) and 13% (n = 14), respectively.

Overall, hospital size had no significant impact on the total number of ABHR-Ds per room (*p* = 0.31). However, in the subset of hospitals with > 500 beds provided more dispensers within one-meter radius of the bed (*p* = 0.002).

**Fig. 1:**
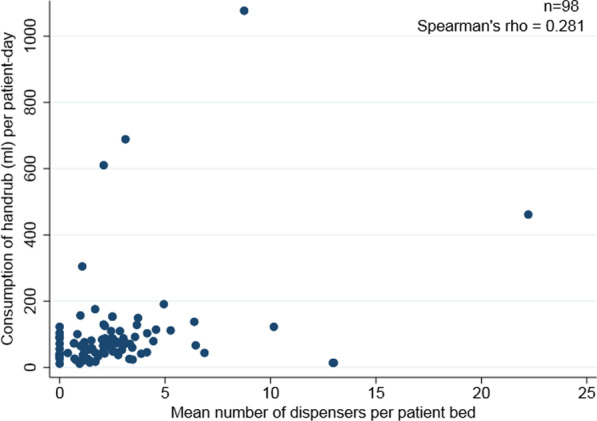
Correlation between the number of dispensers per patient bed and handrub consumption per patient-day (n = 98 hospitals)

**Fig. 2:**
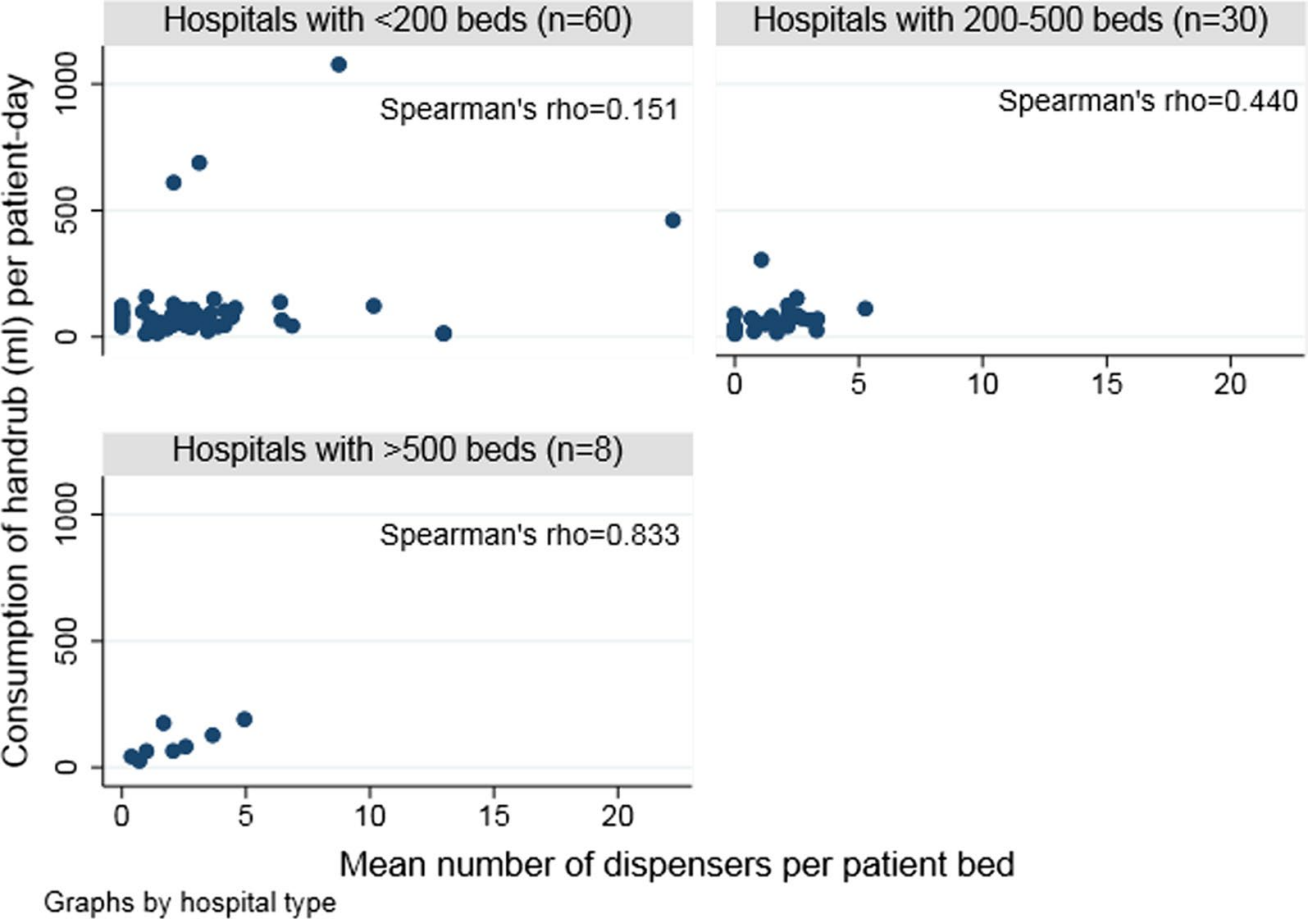
Correlation between the number of dispensers per patient bed and handrub consumption in milliliter per patient day by hospital size

Similarly, the mean number of ABHR-Ds per patient bed did not correlate with handrub consumption (ρ = 0.28) (Fig. 1). However, in large hospitals with more than 500 beds, handrub consumption correlated significantly with the number of ABHR-Ds per patient bed (ρ = 0.83) (Fig. 2).

## Discussion

The importance and effectiveness of HH to prevent healthcare associated infections has been documented in several studies [17, 20]. However, compliance remains an ongoing challenge. Not only do factors such as workload, visibility and location of ABHR-Ds contribute to HH compliance, but also a good skin tolerance of the product, and the instruction of the healthcare workers [9, 12, 13, 21]. To our knowledge, this is the first study published to assess the number and location of ABHR-Ds in a national survey.

Up to now, only the RKI issued recommendations on the number of ABHR-Ds, namely one ABHR-D per bed in intensive care units and 0.5 ABHR-D per bed in general wards as minimum requirement [2]. Our data suggests that the current number in Switzerland is 2–4 times higher with a mean of 2.4 ABHR-Ds per patient bed in large acute care hospitals. One small study also suggested two dispensers per patient bed, and higher number did not result in improved compliance regarding the use of handrub [8]. Therefore, 0.5 dispenser per patient bed as standard –suggested by the German public health service RKI—appears to be too low as minimal standard given the higher number of ABHR-Ds in Switzerland. Importantly, these wall-mounted dispensers are provided in addition to the pocket dispensers in most hospitals. Surprisingly, the introduction of wearable pocket-sized dispensers failed to increase HH compliance or ABHR consumption in an emergency department already well equipped with mounted dispensers [22]. Therefore, hospitals may not improve compliance by additionally offering pocket-size dispenser, if sufficient number and optimized location of ABHR-Ds are available.

Wall-mounted dispensers were the dominant type of ABHR dispensers in 75% (n = 82) of hospitals compared to 25% where pocket-sized dispensers are mainly offered. However, 97% (n = 107) hospitals provide pocket-sized dispensers as an additional tool to wall-mounted dispensers to facilitate access to handrub. Overall, the reported mean number was two ABHR-Ds per patient room (mean: 1.83, range 1–4). Out of 110 hospitals, 91 (83%) provided detailed data on the total number of beds and dispensers within the building, not only in patient rooms, resulting in a higher mean of 2.4 (range 0.4–22.1) ABHR-Ds per hospital bed. Therefore, the number of ABHR-Ds per bed is higher than the number of ABHR-Ds per room since fixed mounted dispsenser e.g., on mobile care trolleys were also included. Data regarding the availability of disinfectant dispensers in European countries was published in 2015. It showed that accessibility to dispensers varied greatly between countries, and there was also a variance between ICU, medical and surgical wards. It should be noted that in this study, 100% availability was achieved in all departments in Switzerland [6].

In contrast to several other studies that highlight the importance of the positioning of ABHR-Ds at the point-of-care to improve HH compliance [4, 5, 13, 15, 17, 23], our study revealed that ABHR-Ds are frequently located at the entrance of the patients’ room and near the sink. Hence, these locations reflect the workflow in a patient room, which plays an important role in HH compliance [15]. As demonstrated in former trials, the visibility of ABHR-Ds is another important factor for HH compliance [3, 4, 18]. This fact is taken into account, reflected by ABHR-Ds located within one-meter radius form the bed in 28% of hospitals and at the bedside in 24%, respectively. Along with the wide usage of pocket dispensers, at least one ABHR-D is available at the point of care in the majority of patient beds. This finding is consistent with the observation in the PROHIBIT study, where a correlation between availability of ABHR and consumption was observed [6].

Small hospitals reported more frequently to have bed-mounted ABHR-Ds, whereas large hospitals tend to prefer a location within one-meter radius from the bed probably due to organizational reasons. Our data demonstrates a positive correlation between number of ABHR-Ds per bed and handrub consumption, statistically significant in hospitals with more than 500 beds. Similar results were seen in two European studies, suggesting that there are more HH opportunities in larger hospitals or possibly, monitoring of compliance is more accurate [6, 7]. Most large hospitals are staffed with board-certified trained infection control practitioners, while smaller hospitals commonly have a dedicated nurse without federally regulated formal training. Therefore, we hypothesized that data quality may be lower in smaller hospitals.

Our study has several limitations. As our survey was conducted anonymously, reported data could not be validated on site. However, most individuals asked to respond were participants in the network Swissnoso for more than 5 years. Out of 110 hospitals, only 91 (83%) were able to respond on the detailed number of dispensers in their hospital, which may lead to an over- or underestimation of the mean ABHR-Ds per patient bed. We found a statistically significant correlation between handrub consumption and the number of dispensers per patient bed only in hospitals with more than 500 beds. In the PROHIBIT study [6] the authors report that “higher AHR consumption in University hospitals may be due to both having an academic attitude towards patient safety, and having a larger budget compared with general hospitals”. Smaller hospitals patients generally suffer from few comorbidities, fewer immunocompromised patients and fewer ICU beds, all partly explain the lower number of opportunities for hand hygiene.

The type of hospitals—large versus small, paediatric versus adult—have not been evaluatated in detail, but influences the study results as observed in the PROHIBIT study [6]. However, the quality of data from larger hospitals in Switzerland commonly excels those from smaller hospitals, since the latter still do not use electronic patient charts, or similar computer-based data: Such analyses are very difficult, since there is commonly a collinearity between size of hospitals, number of infection control staff, quality improvement programs and other factors [6]. Paediatric beds are below 5% of all Swissnoso hospitals, and therefore, do not seriously influence the main results: However, a similar study should be done focusing on paediatric institutions where results might differ from this study in adult hospitals.

## Conclusions

In conclusion, Swiss acute care hospitals offer on average 2.4 permanently wall-mounted dispensers per bed in addition to pocket dispensers in one fourth of hospitals. In large hospitals, consumptions correlated with the number of dispensers suggesting improved compliance with the number of dispensers available. These data could provide a guidance for developing a minimal standard for ABHR-Ds per patient bed, serving as guide for renovating or construction of new hospitals. A standard currently lacking in most countries.

## Supplementary Information

**Additional file 1**. Original Questionnaire in German, French and Italian.

## Data Availability

The datasets used and/or analysed during the current study are available from the corresponding author on reasonable request. All data generated or analysed during this study are included in this published article.
