# Supplementary material for: Handrub dispensers per acute care hospital bed: a study to develop a new minimum standard
Source: Antimicrob Resist Infect Control. 2021 Jun 16;10:93. doi: 10.1186/s13756-021-00949-0 (PMC8206889; doi:10.1186/s13756-021-00949-0)

**Supplementary appendix 1 – 3**

**Appendix 1 (Online questionnaire in Italian)**


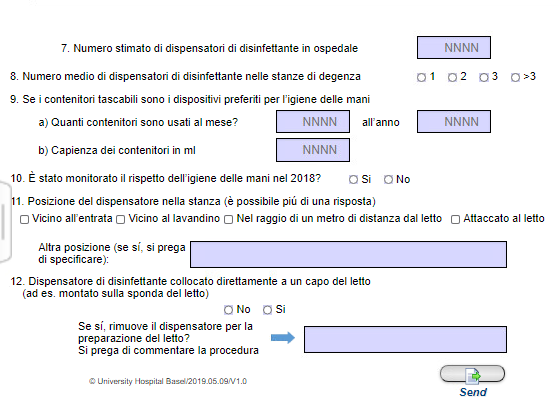

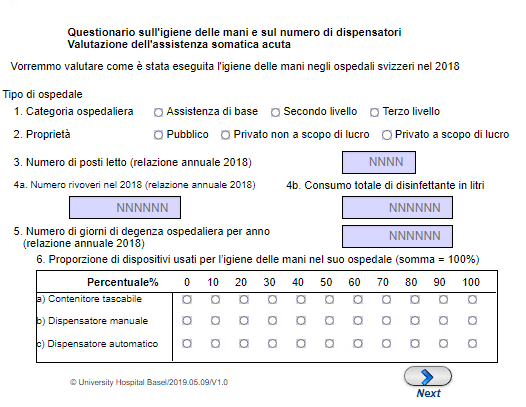


**Appendix 2 (Online questionnaire in French)**


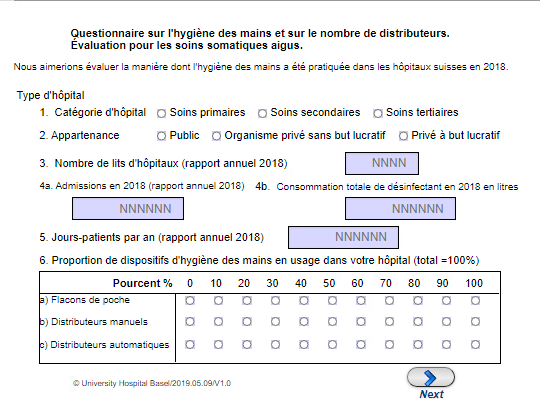


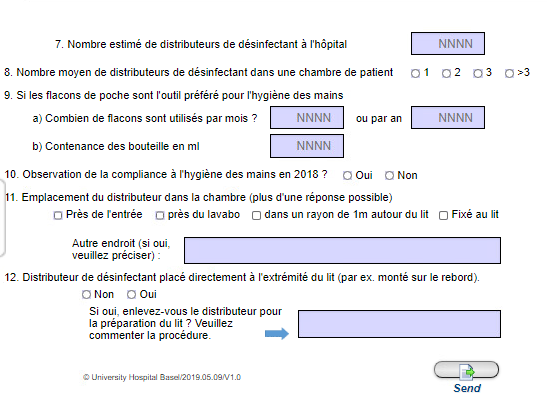


**Appendix 3 (Online questionnaire in German)**


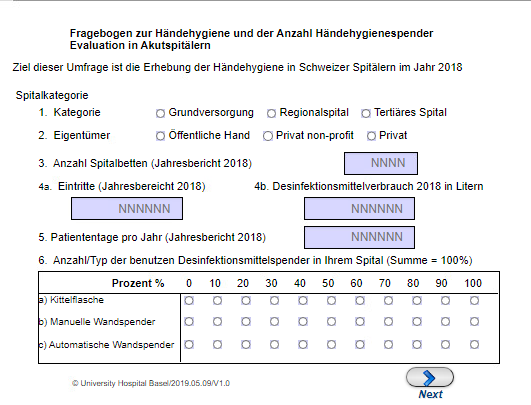


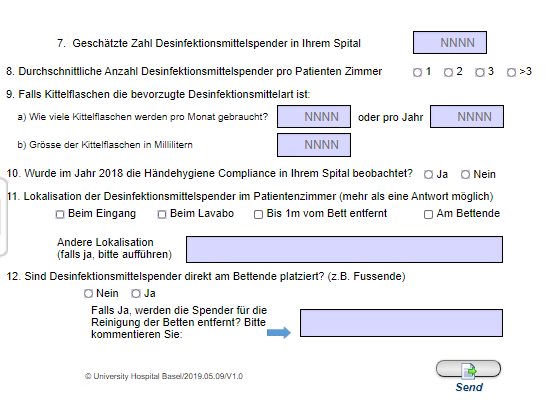

Supplement: Supplementary file 1 — Additional file 1. Original Questionnaire in German, French and Italian. [file 13756_2021_949_MOESM1_ESM.docx]
